# Management of Periprosthetic Joint Infections: A Multidisciplinary Approach

**DOI:** 10.3390/antibiotics15060614

**Published:** 2026-06-17

**Authors:** Madhan Jeyaraman, Filippo Migliorini, Luise Schäfer, Naveen Jeyaraman

**Affiliations:** 1 Department of Regenerative Medicine, Agathisha Institute of Stem Cell and Regenerative Medicine (AISRM), Chennai 600030, Tamil Nadu, India; 2 Department of Orthopaedics, Dr. MGR Educational and Research Institute, ACS Medical College and Hospital, Chennai 600077, Tamil Nadu, India; 3 Orthopaedic Research Group, Department of Orthopaedics, Coimbatore 641045, Tamil Nadu, India; 4 Department of Trauma and Reconstructive Surgery, University Hospital of Halle, Martin-Luther University Halle-Wittenberg, 06097 Halle (Saale), Germany; 5 Department of Orthopaedic and Trauma Surgery, Eifelklinik St. Brigida, Kammerbruchstr. 8, 52152 Simmerath, Germany; 6 Department of Life Sciences, Health, and Health Professions, Link Campus University, Via del Casale di San Pio V, 00165 Rome, Italy

**Keywords:** periprosthetic joint infection, arthroplasty, biofilm, diagnosis, revision surgery, antimicrobial therapy, prevention, multidisciplinary management

## Abstract

Periprosthetic joint infection (PJI) remains one of the most severe and complex complications following joint arthroplasty. With the global increase in primary hip and knee replacements, the clinical and economic burden associated with PJI continues to grow. Although relatively uncommon, PJI is linked to substantial morbidity, elevated mortality, and significantly higher healthcare costs compared to aseptic revision procedures. The challenge is compounded by the intricate pathogenesis of biofilm-forming microorganisms, heterogeneous clinical presentations, and the lack of universally standardised diagnostic criteria. This review provides an integrated overview of current evidence concerning the pathophysiology, risk factors, and microbiological patterns associated with PJI. Contemporary diagnostic pathways based on the Musculoskeletal Infection Society/International Consensus Meeting (MSIS/ICM) criteria are summarised, including the utility and limitations of established serological markers, emerging synovial biomarkers such as alpha-defensin, and the complementary roles of culture techniques, histopathology, and molecular assays. Medical and surgical treatment strategies are outlined, including debridement with implant retention, one-stage and two-stage revision approaches, and organism-directed antimicrobial therapy. Preventive strategies spanning preoperative optimisation, intraoperative protocols, and postoperative risk reduction are also highlighted. Despite significant advances, important gaps persist, particularly in antimicrobial resistance, the management of polymicrobial or culture-negative infections, and the treatment of high-risk or immunocompromised patients. Continued interdisciplinary collaboration and high-quality clinical research are essential to refine diagnostic algorithms, improve therapeutic outcomes, and reduce the incidence of this increasingly consequential complication.

## 1. Introduction

With an increasing ageing population and the current obesity epidemic, the number of joint replacement operations performed annually has also increased considerably, and these now form an important part of elective orthopaedic practice. This is highlighted by projections indicating that by 2060, total hip replacement (THR) and total knee replacement (TKR) will increase by 37.7% and 36.6%, respectively, from their 2018 baseline values [1]. Importantly, most of the predicted arthroplasty procedures will be on geriatric patients, who are known to have higher perioperative risk and comorbidity [1].

This uptick in arthroplasty volume has likewise led to an increase in the absolute number of revisions, which are performed in about 10% of total knee arthroplasty (TKA) cases for multiple reasons such as aseptic loosening, instability, and infection. Periprosthetic joint infection (PJI) is one of the most worrisome complications of arthroplasty with both clinical and economic implications. PJI is defined as an infection of the joint prosthesis and surrounding tissues [2]. The total incidence of PJI is 1–2% [2], but no uniform definition is currently accepted in the literature [3]. PJI has been implicated as the primary indication for revision surgery, with single-centre studies in India finding PJI to cause 38% of revision surgery [4]. Notably, it has also been observed that while aseptic loosening was implicated as the most common cause of revision surgery in the Western world, infections lead the way in India [5,6].

PJI poses a significant mortality burden on the patient that extends far beyond the postoperative period, with a mortality rate of 8% [5] and a relative five-year survival rate of 87.3% [6]. Beyond the clinical implications, PJI also exacts a severe financial toll. The projected 2030 financial burden of PJI was estimated at $1.85 billion [6]. Kurtz et al. [7] observe that the annual cost for revision surgeries post-infection rose from $322 million to $566 million. The financial cost for revision surgeries for PJI is exponentially higher than for their non-PJI counterparts [7]. Unfortunately, the figures quoted above are a gross underestimate, as the current literature has not covered the indirect costs. All these issues are further magnified in patients with multiple arthroplasties; there is a 15% increased risk of a second site of a pre-existing prosthetic joint suffering from PJI if a single site has undergone PJI [8].

The purpose of this narrative review is not to replace guidelines but to critically examine existing strategies and highlight areas where further studies and consensus are needed regarding PJI pathophysiology, risk stratification, diagnostic criteria, and multidisciplinary management.

## 2. Materials and Methods

A narrative review of the current literature on periprosthetic joint infection (PJI), with an emphasis on its pathophysiology, diagnosis, and multidisciplinary management, was conducted. Electronic databases such as PubMed/MEDLINE, Scopus, and Google Scholar were thoroughly searched for relevant articles using the following keywords: “periprosthetic joint infection”, “arthroplasty infection”, “biofilm”, “revision surgery”, “antimicrobial therapy”, and “PJI diagnosis”.

Published studies were eligible for inclusion if they appeared in English (no date restriction was imposed), to allow both foundational and contemporary evidence to be included. Randomised controlled trials, prospective cohort studies, systematic reviews, meta-analysis, and guidelines (particularly from the Musculoskeletal Infection Society (MSIS), International Consensus Meeting (ICM), and the European Bone and Joint Infection Society (EBJIS)) were prioritised as primary sources. The data were narratively synthesised and key findings regarding pathogenesis, diagnostic criteria, surgical approach, antimicrobial therapy, and prevention were incorporated and presented as a composite synopsis with clinical application.

## 3. Prosthetic Joint Infection (PJI)

### 3.1. Pathogenesis of PJI

The major challenge in treating PJI stems from its complex pathogenesis. PJIs may occur at any time following arthroplasty and are commonly classified by onset as early (≤3 months), delayed (3 months–2 years), or late (>2 years) infections [2]. The microorganism reaches the implant site through three major pathways. First, it can be inoculated intraoperatively through an aseptic surgical technique. Second, it reaches the joint site through infection of surrounding regions, such as a superficial skin infection at the operative site, traumatic inoculation of the microorganism into the joint, or infection of the surrounding fascial planes. Lastly, it can reach the joint site via direct hematogenous spread, which is rare, and patients with this mode of spread are noted to have severe bacteraemia [9]. Irrespective of the mode of transmission to the joint, once the microorganism reaches the joint, it forms a biofilm.

In delayed and late-onset PJI, the biofilm will greatly reduce the diagnostic sensitivity of standard synovial fluid and periprosthetic tissue cultures and require the use of additional diagnostic tools (such as sonication). Biofilms are structured, sessile communities of microorganisms embedded in a self-produced extracellular polymeric substance (EPS) matrix that strongly adheres to prosthetic surfaces [10]. Biofilm formation occurs in four sequential stages: initial reversible adhesion of the planktonic bacteria to the implant surface, irreversible attachment mediated by surface adhesins (e.g., microbial surface components recognising adhesive matrix molecules, or MSCRAMMs), micro-colony formation and matrix elaboration, and biofilm maturation and dispersal of daughter cells to seed new infection foci [11]. This organised architecture provides multiple survival advantages: a low bacterial inoculum is sufficient to establish infection owing to the protective environment; the EPS matrix physically shields bacteria from phagocytic clearance and opsonisation; the metabolic rates of bacteria within the biofilm are slow, and thus conventional antibiotics which target active cell processes are largely ineffective; horizontal gene transfer can occur within the biofilm community, allowing rapid acquisition and dissemination of antibiotic resistance determinants [2].

Initially, the infection is confined to the joint space. PJI can then spread to the adjacent bone and soft tissues, causing severe joint damage. If untreated, PJI can spread systemically, leading to systemic manifestations of PJI that have a poor prognosis compared to localised infections [12].

### 3.2. Risk Factors

The risk factors for PJI can be broadly categorised into modifiable and non-modifiable risk factors. Modifiable risk factors include patient and preoperative factors, whereas non-modifiable factors include genetic and gender factors [13]. Patient-related risk factors include male sex [14]; body mass index [15,16]; corticosteroid therapy [16]; suffering from diabetes mellitus [15,16], hypoalbuminemia [16], rheumatoid arthritis [17], malignancy [16]; immunosuppression [16]; poor nutrition [18,19] and drug and tobacco use [14,20]. Surgical factors include increased surgical time [14]; non-same-day surgery [14]; revision surgery [14,21]; wound dehiscence [16]; presence of a drain in the wound [16]; National Nosocomial Infections Surveillance Score ≥ 2 [16].

### 3.3. Microorganism-Implicated

Bacteria and fungi are the most commonly implicated organisms causing PJI [22,23]. The causative microorganisms vary depending on the route of infection and the time interval between surgery and symptom onset. Small sample sizes and single-institutional designs limit most studies on the etiological agents of PJI. Still, Benito et al. [24] reported that *S. aureus*, streptococci, gram-negative bacteria, and enterococci are the most commonly causative organisms of PJI. On the other hand, Patel et al. [25] note *C. acnes* as the causative agent in 44% of shoulder PJI. Tai et al. [26] note that 70% of PJI are monomicrobial and 30% are polymicrobial. This is of significant interest, as polymicrobial populations are increasingly resistant to conventional antimicrobials compared with their monomicrobial counterparts [27]. To further complicate existing treatment protocols, a significant increase in methicillin-resistant *S. aureus* (MRSA) has been observed in infected periprosthetic joints, associated with a decrease in coagulase-negative staphylococci (CoNS) [28].

The clinical implications of pathogen identity extend well beyond microbiological classification. *Staphylococcus aureus* is the most virulent of the common PJI pathogens, characterised by robust biofilm formation, toxins production, and an aggressive early clinical course; infection with *S. aureus* is associated with higher rates of treatment failure and mortality compared with CoNS. Coagulase-negative staphylococci, particularly *Staphylococcus* epidermidis, tend to cause more indolent, low-grade infections, often presenting as delayed or late PJI. Infections with these organisms may be missed or diagnosed late due to the slow growth rate (cultures should be held for a minimum of 14 days) and the low virulence of CoNS. Culture-negative PJI, defined as infection meeting diagnostic criteria but in the absence of a positive culture, presents a major management challenge; it may reflect prior antibiotic exposure, fastidious or slow-growing organisms, biofilm-encapsulated organisms, or inadequate culture technique and is increasingly addressed using molecular diagnostic tools and prolonged culture protocols; it is most common with the PJI organisms associated with shoulder arthroplasty.

### 3.4. Classification of PJI

The classification by Coventry et al. [29] is the most widely accepted, but the most common classification in the literature on PJI is that of Tsukayama et al. [30]. Other classifications include those given by Toms et al. [31], Cui et al. [32], Zimmerli et al. [33], Parvizi et al. [34], Osmon et al. [35], and Romano et al. [36,37]. A new classification by Pellegrini et al. [38] was given to encompass cases that do not fit the currently available classification. Table 1 shows the classification of PJI according to the Coventry, Tsukayama, and Pellegrini classifications.

### 3.5. Diagnosis

PJI confined to the joint presents pain, erythema, and swelling around the infected joint [39]. The pain is persistent and progressively worsens. Systemic manifestations are absent in such cases. Disseminated PJI can show systemic manifestations and bacteraemia. Tokarski et al. [12] demonstrated that patients with disseminated PJI fulfilling three or more of the systemic inflammatory response syndrome (SIRS) criteria had worse outcomes. The trifecta of a high economic burden, poor outcomes, and diagnostic challenges makes PJI a modern-day orthopaedic Pandora’s box. Hence, clinicians should be alert and follow a stepwise protocol to accurately and timely diagnose PJI.

#### 3.5.1. History and Clinical Examination

Patients with PJI usually present in the outpatient Orthopaedic department, where a detailed history and clinical examination should be undertaken. The typical symptoms mentioned above suggest that PJI should be followed up on with imaging and serological markers. The diagnostic quandary arises in those PJI cases where clinical symptoms are absent, and a high degree of suspicion is required. This is especially seen in late-onset PJI [40].

#### 3.5.2. Radiographic Imaging

Plain radiographs can reveal the wide band of lucency suggestive of bone destruction and are therefore the most common imaging modality suggested for PJI [41]. However, their sensitivity and specificity are poor [42], as they cannot distinguish accurately between aseptic loosening and infection. Bone scintigraphy with 99mTc shows high sensitivity but low specificity for PJI [43]. Similarly, computed tomography (CT) and magnetic resonance imaging (MRI) provide limited low specificity in this context. As such, imaging does have a place in the workup of PJI as an adjunct to other clinical data, allowing for anatomical detail, evaluation of implant loosening or periprosthetic osteolysis, excluding other diagnoses and aiding in surgical planning; however, it is not presently recommended as a standalone diagnostic tool [43].

#### 3.5.3. Serological Markers

Multiple serological markers can diagnose PJI [44], each possessing particular strengths and limitations (Table 2).

#### 3.5.4. Synovial Markers

The aspiration of synovial fluid and the testing of the aspirate for specific markers are currently the novel diagnostic breakthroughs proposed by orthopaedic researchers. The procedure can be performed as an outpatient for infected knee arthroplasty or under fluoroscopic guidance for hip arthroplasty. Table 3 highlights the synovial markers currently used.

#### 3.5.5. Culture and Histopathological Examination

When synovial fluid is negative for PJI, then intraoperative frozen-section sampling or biopsy and culture can be performed. The histologic criteria for PJI are defined as the presence of more than five neutrophils per high-power field in at least five fields at a magnification of ×400 [45]. Yet, the current literature states that the above criteria are too high and should be reduced [46]. Furthermore, the sectioning should not be done using cautery [47]. Tissue culture, on the other hand, has low sensitivity [48]. A culture-negative PJI indicates that the microorganism colonising the joint is either fungal, mycobacterial, or Coxiella in nature [48].

Sonication of explanted implants represents a valuable adjunct for microbiological confirmation of PJI, especially in chronic or low-grade infections driven by biofilm-forming organisms. By mechanically disrupting the biofilm and releasing bacteria into the sonication fluid, this technique can increase the diagnostic yield compared with standard periprosthetic tissue cultures. In a pivotal prospective study, Trampuz et al. [49] demonstrated a substantially higher sensitivity of sonication fluid culture compared with periprosthetic tissue culture (78.5% vs. 60.8%), while maintaining excellent specificity (98.8% vs. 99.2%). The benefit was particularly relevant in patients receiving antibiotics prior to revision surgery, in whom sonication demonstrated higher sensitivity than conventional tissue sampling [49]. Consistent evidence from subsequent, prospective cohorts further supports the superiority of sonication. Tani et al. [50] reported significantly higher sensitivity for sonication fluid culture compared with tissue cultures (77.04% vs. 55.73%, *p* = 0.012), with high specificity for both methods. In a larger revision cohort, Banousi et al. [51] confirmed markedly improved sensitivity of sonication over tissue cultures (91% vs. 43%, *p* < 0.005), while also highlighting the added microbiological value of sonication for detecting phenotypic heterogeneity relevant to antimicrobial susceptibility interpretation. Beyond diagnostic yield, clinical outcome data suggest a potential therapeutic impact. In revision arthroplasty patients, systematic sonication helped detect occult infections and guided earlier targeted antibiotic therapy, which correlated with improved Oxford hip/knee scores compared with cases in which sonication was not performed [52]. Overall, sonication is a reliable complementary method within the diagnostic workflow for PJI, particularly in suspected culture-negative infections and in revision settings where biofilm-related bacterial persistence is likely.

#### 3.5.6. Molecular Method

Innovative molecular methods, such as multiplex polymerase chain reactions, are being proposed to diagnose PJI, but large-scale studies have yet to be conducted.

#### 3.5.7. Current Algorithm and Criteria

The diagnosis of PJI follows a structured approach defined by the Musculoskeletal Infection Society (MSIS) and refined by the ICM [43,45,53]. These criteria integrate clinical, serological, microbiological, and histopathological findings to ensure accurate identification of infection [43,53].

The diagnostic process begins with a preoperative evaluation, during which serum inflammatory markers, such as C-reactive protein (CRP) and D-dimer, are assessed. If these values are elevated, synovial fluid analysis is recommended to determine the leukocyte count, percentage of polymorphonuclear neutrophils (PMN%), and to perform culture and biomarker testing (e.g., leukocyte esterase or alpha-defensin) [54].

According to the updated MSIS/ICM criteria, a diagnosis of PJI is confirmed when at least one major criterion is present: (1) a sinus tract communicating with the prosthesis or (2) two positive cultures of the same microorganism, or when a combination of minor criteria yields a diagnostic score ≥ 6 [43,53]. Minor criteria include elevated CRP or D-dimer, increased synovial WBC count or PMN%, positive histology, and one positive culture [43,53].

The MSIS diagnostic algorithm provides a stepwise framework for evaluating suspected PJI cases, beginning with clinical suspicion and laboratory screening, followed by synovial fluid testing and, where necessary, intraoperative confirmation. This systematic approach helps distinguish acute from chronic infections and guides medical and surgical management strategies [55]. Nevertheless, it is important to acknowledge that diagnostic criteria vary across societies and regions. In addition to the widely used MSIS/ICM criteria, alternative diagnostic frameworks have gained increasing acceptance, particularly in Europe. The European Bone and Joint Infection Society (EBJIS) definition proposes a pragmatic, likelihood-based classification (infection unlikely, likely, confirmed) that integrates clinical findings, serum and synovial biomarkers, histology, microbiology, and intraoperative observations into an overall diagnostic interpretation [56]. In contrast to the score-driven MSIS/ICM approach, the EBJIS framework explicitly supports clinical decision-making in borderline or low-grade infections where individual tests may remain inconclusive.

Importantly, there is no universally agreed diagnostic criterion, meaning prevalence and treatment outcomes of PJI may not be comparable between centres, depending on the context of the tests performed and the experience in the specific setting. The need for clear numerical thresholds offsets the simplicity of the MSIS/ICM of the need for a scoring system. At the same time, the more flexible, likelihood-based framework of the EBJIS better reflects diagnostic uncertainty. It allows for contextual diagnosis, a factor that may be particularly important in culture-negative PJI, PJI following antibiotic use, and PJI where only some diagnostic markers have been performed.

### 3.6. Management Strategies and the Challenge Posed

The treatment for PJI is multidisciplinary and requires individualised care for each patient. The main goal of treatment is to alleviate suffering at a fractional cost to the patient and prevent future complications. Treatment strategies can be divided into medical and surgical approaches. Challenges arise in managing immunocompromised patients, in whom Ji et al. [57] reported multiple recurrences despite appropriate medical and surgical therapies. The authors further state that the main treatment goal in such patients should be to limit disease activity rather than achieve complete disease cessation [57]. The second challenge arises in patients with significant bone loss, for whom the custom-made articulating spacer (CUMARS) construct has proven effective [58].

#### 3.6.1. Medical Management

Medical management should be administered concurrently with surgical management. Only medical management should be avoided if the patient does not consent to surgery or if surgery poses a significant health risk [59]. Antimicrobials form the mainstay of medical management. Most antimicrobials are administered after culture confirmation, but Benito et al. [24] have proposed guidelines for empirical antimicrobials. Their recommendations are summarised in Table 4. Rifampicin and quinolones should not be given empirically or as first-line drugs. The major challenge remains the increasing prevalence of antibiotic resistance, with early postoperative PJI demonstrating resistance to cefazolin [60]. Casenaz et al. [61] also found that a vancomycin and cefotaxime combination decreased effectiveness in early and late PJIs.

Empirical therapy should be de-escalated to targeted, organism-directed antimicrobial therapy once microbiological results are available; however, the duration of antimicrobial therapy depends on the surgical strategy used: 3–6 months of targeted oral antimicrobial therapy is provided following DAIR, 3 months following one-stage revision, and 4–6 weeks of targeted therapy between stages/indeterminate duration following reimplantation in two-stage revision; oral step-down therapy is the transition from an initial course of parenteral therapy to oral agents and is now widely accepted, as it decreases catheter-related morbidity without compromising outcomes, provided the bioavailability is adequate. However, for those in which surgery is not feasible because of excessive operative risk, patient refusal, or advanced comorbidity, indefinite suppressive oral antimicrobial therapy may be appropriate, although this does not result in a cure and carries long-term risks of resistance and adverse effects.

#### 3.6.2. Surgical Management

Surgical management of PJI involves several approaches that are selected based on the timing of infection, the stability of the prosthesis, and the condition of the surrounding soft tissue. Various treatment algorithms have been proposed to guide clinical decision-making. Among them, Li et al. [59] suggested a framework categorising procedures into debridement with implant retention, one-stage exchange, and two-stage exchange surgery. In accordance with these principles, the main surgical strategies and their reported success rates are summarised in Table 5. The use of antimicrobial therapy should always be in combination with surgical treatment; medical therapy alone is not enough to cure the vast majority of PJIs and is only appropriate for a patient for whom surgical intervention is deemed to have an unacceptable risk or when the patient refuses to have surgery.

The choice of surgical strategy depends on many interdependent factors. The rates of success decrease significantly if these factors are not observed: DAIR is best suited to acute infections (those of onset within 3–4 weeks of arthroplasty, or within 3 weeks of haematogenous seeding) with a stable, well fixed arthroplasty, adequate soft tissue envelope, and a known and susceptible organism; two-stage revision is best suited to chronic infections, sinus tracts, resistant organisms (including MRSA and fungi), significant bone loss, and when the causative organism is unknown, as this procedure allows for thorough debridement, the placement of an antibiotic loaded cement spacer, and interval confirmation of eradication of infection prior to reimplantation; patient-level factors (immunocompromise, nutritional status, functional demands, and tolerance of a second procedure) are also important in determining strategy selection.

### 3.7. Multidisciplinary Team-Based Management

Effective management of PJIs is truly multidisciplinary and relies on the coordinated efforts of several clinical and allied health specialities. The orthopaedic surgeon should assume the lead in surgical decision-making, considering the indication(s) for surgery, the chronicity of the infection, the stability of the prosthesis, and the bone and soft tissue quality. The infectious diseases physician or clinical microbiologist is crucial in the interpretation of results, the identification of the causative organism and its susceptibility profile, as well as the selection of targeted antimicrobial therapy, including route, length of antimicrobial treatment, and selection of biofilm-active drug. The clinical microbiologist must ensure optimal handling of specimens and culture protocols, adequate incubation periods (especially in the case of slow-growing organisms, such as *C. acnes*), and the use of molecular testing when indicated. The radiologist will give an anatomical assessment and will perform image-guided aspiration of the joints, especially for hip prostheses, which require fluoroscopy. Plastic and reconstructive surgeons may be required when there are substantial soft tissue defects that may not allow for the wound to be closed, or when local flaps are required to provide sufficient coverage. The pharmacist helps with AMS, monitoring drug levels, drug interactions, and transition to oral antimicrobials. Throughout the treatment pathway, the wound care, mobilisation, and rehabilitation are managed by nursing staff and physiotherapists. Formalised multidisciplinary team (MDT) discussion at diagnosis and at key decision points, especially when choosing surgical strategy, planning the timing of reimplantation in two-stage revision, and the endpoint of antimicrobial therapy are all associated with optimal outcomes.

### 3.8. Prevention

#### 3.8.1. Antibiotic Prophylaxis

The ICM recommends using a first-generation cephalosporin one hour before surgery. In longer surgeries, prophylaxis can be administered two hours before surgery [66]. In high-risk cases, vancomycin and teicoplanin can be administered. These high-risk cases include the following:

1. Carriers of MRSA or patients who are admitted to units that have an outbreak of MRSA
2. Patients in the dialysis unit
3. Healthcare workers
4. Patients allergic to penicillin

The optimal antibiotic type and the duration of antibiotic prophylaxis continue to develop, especially with the increase of antibiotic-resistant organisms, with antibiotic stewardship principles employed to try to minimise selective pressure while maintaining effectiveness for infection prevention [67].

#### 3.8.2. Operative Preventive Methods

Preoperative correction of anaemia should be the priority before the patient is posted for surgery [68]. Management should be achieved through iron and erythropoietin supplementation rather than blood transfusion [69]. Neuraxial anaesthesia should also be preferred in such patients [70]. Preoperative hair removal should be done at the surgery site using clippers or depilatory creams. Skin preparation should be done using a combination of alcohol–CHG antiseptic [71]. Surgeons should practice adequate hand hygiene, and double- or triple-gloving should be performed before operating. Draping should be done using plastic adhesive tape rather than cloth drapes [72]. The operating room (OR) environment should maintain laminar airflow for the duration of surgery and OR traffic. In high-risk patients undergoing elective arthroplasty, antibiotics should be impregnated in the cement [73]. During the surgery, suction tips should be changed every sixty minutes.

#### 3.8.3. Postoperative Preventive Measures

Prophylactic antibiotics before dental procedures, colonoscopy, or endoscopy are debatable and are not recommended in the majority of patients with joint prostheses, but might be considered on an individual basis after multidisciplinary discussion in high-risk patients such as those who are immunocompromised, have a history of PJI, or are undergoing a procedure associated with a high risk of developing bacteraemia. Practitioners should follow the latest local and national guidelines pertinent to their clinical practice [74]. Antibiotics should not be administered if patients have a viral infection.

### 3.9. Future Directives and Research Gap

There are multiple directions for future research. From a manufacturing and biomaterials standpoint, there is increased interest in developing antimicrobial surfaces on implants, which could inherently prevent the formation of biofilms, such as using silver ion coating, titanium dioxide photocatalysis, antibiotic-eluting surface coatings, or developing nano-scale textured implant surfaces that physically repel bacterial adhesion; in addition, there is interest in developing antimicrobial alloys with copper or zinc, which have shown promise in reducing bacterial colonization in animal studies. From a molecular diagnostics standpoint, there is increasing interest in next-generation sequencing (NGS) and multiplex PCR panels applied to synovial fluid or sonication fluid for the diagnosis of culture-negative and low-grade infections. However, more large-scale prospective validation is necessary. Finally, high-quality prospective randomized controlled trials (RCTs) comparing DAIR, one-stage, and two-stage revision in matched patient populations are needed to arrive at more definitive evidence with regard to the selection of the surgical strategy.

## 4. Conclusions

PJI remains a clinically formidable complication of joint arthroplasty, with considerable implications for patient morbidity, mortality, and healthcare expenditure. There are a number of conclusions from the current evidence base that are practical. First, diagnosis should be based on applying structured validated criteria (either MSIS/ICM or EBJIS) and not viewed as set thresholds; when there is diagnostic uncertainty, which in low-grade and culture-negative infections is often the case, this should be investigated systematically, with multidisciplinary input. Second, surgical plans need to be tailored to each patient: there is no single surgical plan that is always the best; decisions should consider the chronicity of the infection, the pathogen type, host factors, and soft tissue status. Third, optimal outcomes can only be achieved using coordinated multidisciplinary decision making, with orthopaedic surgery, infectious diseases, microbiology, radiology, and rehabilitation medicine all playing a role in the decision-making process throughout the treatment journey. Fourth, the growing risk of antimicrobial resistance requires careful antibiotic stewardship at all stages, from prophylaxis to empirical and targeted treatment and suppressive therapy. Ongoing research on high-quality prospective studies, novel diagnostics, antimicrobial biomaterials, and standardised outcome definitions will be key to moving the field forward and enhancing outcomes for this complicated patient population.

## Figures and Tables

**Table 1:** Commonly used PJI classification systems.

Stage/Type	Clinical Presentation	Remarks
PJI presentation according to Coventry et al. [29]		
Type I	Acute postoperative infection	Infection within the first 30 days of surgery
Type II	Late chronic infection	Infection presenting post 30 days of surgery
Type III	Late hematogenous infection	Infection presenting more than 2 years after surgery
PJI presentation according to Tsukayama et al. [30]		
Stage I	≥2 positive intraoperative culture	Positive intraoperative culture
Stage II	Infection within first 30 days of surgery	Early postoperative infection
Stage III	Infection caused by hematogenous dissemination of microorganism in a previously normal non-infected joint	Acute hematogenous infection
Stage IV	Infection present for more than 30 days	Late (chronic) infection
PJI presentation according to Pellegrini et al. [38]		
Type I	Acute postoperative infection	with the infection limited to the joint space
Type II	Acute postoperative infection	with the infection spreading towards the bone prosthesis interface
Type III	Chronic infection	with the infection limited to the joint space
Type IV	Chronic infection	with the infection spreading towards the bone prosthesis interface

**Table 2:** Serological markers for PJI.

Serological Marker	Strengths	Limitation
Erythrocyte sedimentation rate (ESR)	Measured if patients present with pain. Inexpensive, easily available, and widely done. Recommended test by ICM and the AAOS for diagnosing PJI. Normal values do not necessitate the need for joint aspiration	Cannot be used in patients suffering from inflammatory diseases, requiring reimplantation or PJI in the early postoperative period. Shows normal values if PJI caused by *C. acnes* and CoNS
C-reactive protein (CRP)	Measured if patients present with pain. Inexpensive, easily available, and widely done. Recommended test by ICM and AAOS for diagnosing PJI. Normal values do not necessitate the need for joint aspiration	Cannot be used in patients suffering from inflammatory diseases, requiring reimplantation or PJI in the early postoperative period. Shows normal values if PJI is caused by *C. acnes* and CoNS
D-Dimer	89% sensitive and 93% specific. Can diagnose PJI in early postoperative period	Elevated D-dimer levels can be noted in inflammation unrelated to an infective pathology
Interleukin-6 (IL-6)	Can be used to diagnose PJI after primary arthroplasty. Can be used as a marker to distinguish PJI and aseptic loosening. Greater accuracy in chronic PJI	Cannot be used in patients with PJI suffering from concomitant Paget’s disease, immunodeficiency syndrome, or chronic inflammatory disorder
Procalcitonin	Rapid rise and fall of procalcitonin values as compared to CRP.	Extremely low accuracy. Furthermore, studies have been conducted in very small population and hence currently this marker is not recommended.

PJI: periprosthetic joint infection; AAOS: American Academy of Orthopaedic Surgeons; CoNS: coagulase-negative staphylococci; ICM: International Consensus Meeting; CRP: C-reactive protein.

**Table 3:** Synovial markers.

Marker	Strength	Limitation
Alpha-defensin	High level of diagnostic accuracy in selected cases; 97% sensitive and specific with a positive predictive value of 88% and negative predictive value of 99% [42]	Amanatullah et.al [43] states that alpha-defensin is very costly, not always available in resource-limited areas, can be falsely positive in cases of metallosis or inflammatory arthropathy, and therefore may be useful as an adjunct to conventional tests that are clearly positive or negative.
Leukocyte esterase	93.3% sensitivity and 77.0% specificity [44]. Is considered in minor criteria for defining PJI by the International Consensus Group	Lack of precise cutoff values and poor specificity

PJI: periprosthetic joint infection.

**Table 4:** Empirical medical management proposed by Benito et al.

Criteria	Management
Non-hematogenous PJI detected within 30 days of surgery	Antimicrobials targeting *Staphylococcus* species (including MRSA), *Enterobacteriaceae*, and *P. aeruginosa*. No need to include antimicrobial cover for anaerobes and ESBL-producing *Enterobacteriaceae*
Non-hematogenous PJI detected between 30 and 90 days of surgery	Antimicrobials targeting *Staphylococcus* species (including MRSA), and *Enterobacteriaceae*. No need to cover *P. aeruginosa* and replacement of cefepime/ceftazidime to ceftriaxone/cefotaxime
Non-hematogenous PJI detected post 90 days of surgery	Empirical therapy is not critical in this situation. Removal of prosthesis should be done
Hematogenous PJI	Antimicrobials targeting *Staphylococcus* species (including MRSA), *E. coli*, and *Enterobacteriaceae* should be mandated

PJI: periprosthetic joint infection.

**Table 5:** Surgical management options for PJI and their reported success rates.

Procedure	Summary	Success Rate
Debridement with retention	Removal of necrotic soft tissue and bone. Prosthesis is not replaced	35% [62] but can be 80% if specific rigid conditions are met [63]
One stage exchange/Revision surgery (Most common)	Excision of the prosthesis, cement, infected bone and soft tissue and replacing it with a new prosthesis and antibiotic-impregnated cement	80–90% [64]
Two stage implant replacement (Gold standard)	Excision of the prosthesis, cement, infected bone and soft tissue followed by a delay and then reimplantation of the new prosthesis. The delay is variable with literature recommending one week for known organisms and two weeks for unknown organisms, fungi, or enterococci	>90% [33] but increased risk of reinfection as compared to one stage surgery [65].

Note: Reported success rates span ranges of heterogeneous patient groups and are highly dependent on patient selection criteria, pathogen responsible for infection, chronicity of infection, surgical approach, antibiotic treatment, and definition used for treatment success (eradication of infection, prosthesis survival, functional outcome). These numbers are to be used as a guideline only. The above factors combine to give individual patient outcomes.

## Data Availability

All relevant data are included in the article.

## Abbreviations

The following abbreviations are used in this manuscript:

AISRM	Agathisha Institute of Stem Cell and Regenerative Medicine
THR	Total Hip Replacement
TKR	Total Knee Replacement
TKA	Total Knee Arthroplasty
PJI	Periprosthetic Joint Infection
PJIs	Periprosthetic Joint Infections
MSIS	Musculoskeletal Infection Society
ICM	International Consensus Meeting
SIRS	Systemic Inflammatory Response Syndrome
CT	Computed Tomography
MRI	Magnetic Resonance Imaging
ESR	Erythrocyte Sedimentation Rate
AAOS	American Academy of Orthopaedic Surgeons
CRP	C-reactive Protein
CoNS	Coagulase-negative Staphylococci
IL-6	Interleukin-6
PMN%	Percentage of Polymorphonuclear Neutrophils
WBC	White Blood Cell Count
EBJIS	European Bone and Joint Infection Society
CUMARS	Custom-Made Articulating Spacer
MRSA	Methicillin-resistant *Staphylococcus aureus*
ESBL	Extended-Spectrum Beta-Lactamase
OR	Operating Room
CHG	Chlorhexidine Gluconate
PCR	Polymerase Chain Reaction
